# Engineering of papaya mosaic virus (PapMV) nanoparticles with a CTL epitope derived from influenza NP

**DOI:** 10.1186/1477-3155-11-10

**Published:** 2013-04-04

**Authors:** Cindy Babin, Nathalie Majeau, Denis Leclerc

**Affiliations:** 1 Department of Microbiology, Infectiology and Immunology, Infectious Disease Research Center, Laval University, 2705 boul. Laurier, Quebec city, PQ G1V 4G2, Canada

**Keywords:** Papaya mosaic virus (PapMV), Vaccine platform, Rod shape nanoparticles, Influenza

## Abstract

**Background:**

The ever-present threat of infectious disease, e.g. influenza pandemics, and the increasing need for new and effective treatments in immunotherapy are the driving forces that motivate research into new and innovative vaccine platforms. Ideally, such platforms should trigger an efficient CTL response, be safe, and easy to manufacture. We recently developed a novel nanoparticle adjuvant comprised of papaya mosaic virus (PapMV) coat protein (CP) assembled around an RNA. The PapMV nanoparticle is an efficient vaccine platform in which the peptide antigen is fused to the C-terminus of the PapMV CP, leading to nanoparticles presenting surface-exposed epitope. The fusion stabilizes the epitope and improves its immunogenicity. We found recently that C-terminal fusions are not always efficient, depending on the nature of the peptide fused to the platform.

**Results:**

We chose a CTL epitope derived from the nucleocapsid (NP) of influenza virus (NP_147-155_) for this proof-of-concept demonstration. Recombinant nanoparticles harbouring a fusion at the N-terminus were more efficient in triggering a CTL response. Efficacy appeared to be linked to the stability of the nanoparticles at 37°C. We also showed that discs—smaller than nanoparticles—made of 20 subunits of PapMV CP are less efficient for induction of a CTL response in mice, revealing that assembly of the recombinant PapMV CP into nanoparticles is crucial to triggering an efficient CTL response.

**Conclusion:**

The point of fusion on the PapMV vaccine platform is critical to triggering an efficient CTL response. Efficacy is linked to nanoparticle stability; nanoparticles must be stable at 37°C but remain susceptible to cellular proteases to ensure efficient processing of the CTL epitope by cells of the immune system. The results of this study improve our understanding of the PapMV vaccine platform, which will facilitate the design of efficient vaccines to various infectious threats.

## Background

Papaya mosaic virus (PapMV) is a member of the large family of *Flexiviridae* in the genus *Potexvirus*. The virus has a flexuous rod shape of 500 nm in length and 13 nm in diameter [1]. The CP, made mostly of alpha helices [2], is composed of 215 amino acids and has an estimated molecular weight of 23 kDa [3]. We showed previously that non-infectious nanoparticles made of recombinant PapMV CP are similar in shape and appearance to wild-type virus purified from plants [3]. PapMV nanoparticles were used previously as a vaccine platform technology to improve the immunogenicity of a peptide antigen fused to the nanoparticle structure [4-8]. The PapMV vaccine platform can induce a long-lasting memory response to an antigen fused on its surface [4]. Previous studies showed the capacity of PapMV nanoparticles to trigger a CTL response, in both *in vitro* and *in vivo* models, when the CTL epitope was fused to the C-terminus of the CP [6,7,9]. Although PapMV tolerates insertion of several peptides to its C-terminus [4-7,10], a recent study revealed that N-terminal fusion of some peptides is also tolerated [8]. Depending on the nature of the amino acid sequence, some peptides can interfere with the CP assembly or with nanoparticle stability, which can affect their ability to stimulate an humoral response. A modification of the fusion site on the CP can help to resolve this issue. In this study, we compared the efficacy of nanoparticles harbouring fusion of a CTL epitope at either the N- or the C-terminus to trigger a cellular immune response.

The crystalline and highly ordered structure of the nanoparticles is critical to triggering an efficient humoral response, as also reported by many other groups [11-13]. However, it is still unknown if assembly into the highly ordered nanoparticle structure, made of several hundreds of PapMV CP, is more efficient than assembly of a smaller disc-like structure (aggregate of 20 subunits) in triggering the CTL response. Since the mechanisms of induction of humoral and CTL immune responses rely on different immune cells and mechanisms, we also evaluated the importance of highly ordered assembly of recombinant PapMV CP into nanoparticles in triggering a CTL response by comparing the immunogenicity of nanoparticles and discs.

## Results and discussion

### Engineering of PapMV nanoparticles fused to the influenza CTL epitope

**Figure 1:**
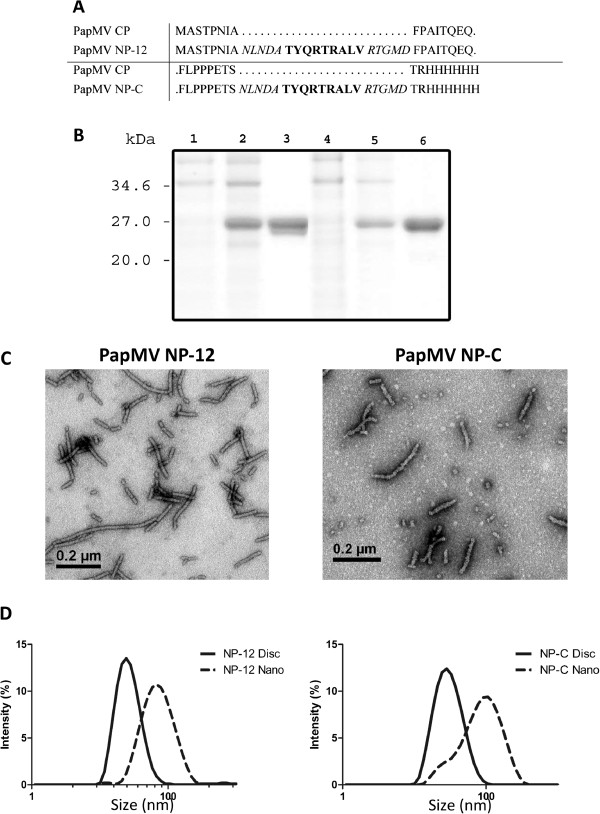
**Recombinant PapMV CP proteins. **(**A**) Amino acid sequences of the N- or C-terminus of the PapMV CP to which fusions were made. The sequence in bold corresponds to the NP_147-155 _epitope from Influenza virus nucleoprotein (NP). The sequences in italics represent the flanking amino acids retained to ensure efficient processing of the epitope. (**B**) Bacterial lysate of the culture before induction with IPTG (first lane), after induction with IPTG (second lane) and after purification with nickel beads (third lane) of PapMV NP-12 (lanes 1–3) and PapMV NP-C (lanes 4–6). (**C**) Transmission electron microscope images of PapMV NP-12 and NP-C nanoparticles, respectively. (**D**) Size of nanoparticles and discs recorded by dynamic light scattering (DLS).

**Figure 2:**
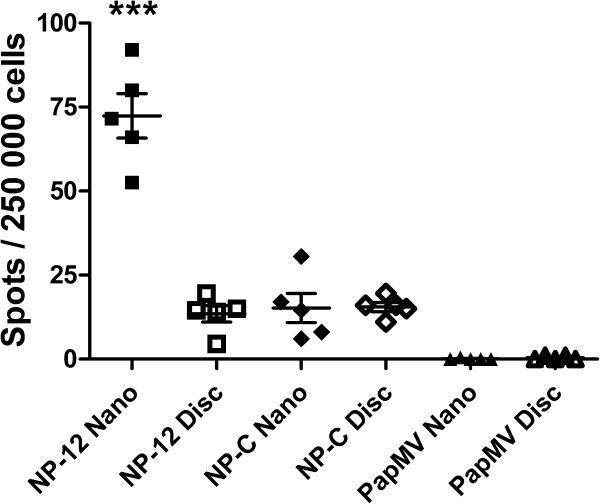
**ELISPOT assays of mice immunized with different forms of nanoparticles. **Mice, 5 per group, were immunized three times by the intraperitoneal route with 100 μg of recombinant PapMV NP-12, PapMV NP-C and PapMV nanoparticles or discs. Two weeks after the second boost, spleens were extracted and ELISPOT assays performed. The precursor frequency of specific T cells was determined by subtracting the background spots in media alone from the number of spots seen in wells reactivated with NP_147-155 _peptide. *** p ≤ 0.001 compared to all groups.

In this study, we used the CTL epitope NP_147-155_, a 9-mer H-2Kd epitope specific for Balb/C mice derived from influenza virus to search for the optimal position for fusion of this epitope to the vaccine platform. The CTL epitope was flanked by 5 native residues of influenza NP protein at both the N- and C-termini to favour natural processing of the peptide, and was fused genetically to either the N- (before F13) or C-terminus of PapMV CP [Figure 1A]. Following expression in *E. coli*, recombinant PapMV particles harbouring the peptide on their surface (named PapMV NP-12 and PapMV NP-C) were affinity purified on a Ni^2+^ column via the 6xHis tag located at the C-terminus [Figure 1B]. Purified proteins were subjected to ultracentrifugation to pellet the nanoparticles. The lower molecular weight forms, composed of discs (20 subunits of CP) remained in the supernatant. As shown by dynamic light scattering (DLS), two structures were obtained with this purification protocol: (1) typical long rod-shaped structures of approximately 90 nm in length for PapMV NP-12 and PapMV NP-C [Figure 1C–D], and (2) smaller discs of approximately 20 nm in length for PapMV NP-12 and PapMV NP-C [Figure 1D]. The DLS method provides a global view of the size of the particles in solution that fits our qualitative observations by electron microscopy, and revealed the difference in size between discs (black line) and nanoparticles (dotted line) for both constructs (Figure 1D). It also shows that structures formed by NP-12 and NP-C constructs are similar in size, confirming observations by transmission electron microscopy showing that PapMV NP-12 and NP-C nanoparticles are similar in length, structure and appearance [Figure 1C].

We previously reported that residue F13 of PapMV CP is critical for the interaction between the PapMV CP subunits when assembling into nanoparticles [14]. We showed that this hydrophobic residue fits snugly inside the hydrophobic pocket of the neighbouring CP [2]. Interestingly, insertion of the NP_147-155_ epitope just before F13 in the N-terminal fusions clearly does not interfere with the interaction between PapMV CP monomers that is crucial for self-assembly of nanoparticles.

### PapMV NP-12 nanoparticles are better inducers of the CTL response

To evaluate the potential of PapMV nanoparticles to induce a CD8+ mediated cellular response, we immunised 6- to 8-week-old Balb/C mice three times at 2-week intervals by the intraperitoneal route with 100 μg of PapMV (without fusion), NP-12 or NP-C nanoparticles. Two weeks after the second boost, spleens were harvested and ELISPOT assays using the NP_147-155_ peptide were performed to quantify the level of IFN-γ secreted by CD8+ cells [Figure 2]. Secretion of IFN-γ is proportional to the level of precursors of CD8+ cytotoxic lymphocytes specific to the fused CTL epitope in vaccinated mice. The result showed that, compared to all the other treatments, mice immunized with PapMV NP-12 nanoparticles secrete significantly more IFN-γ [Figure 2].

**Figure 3:**
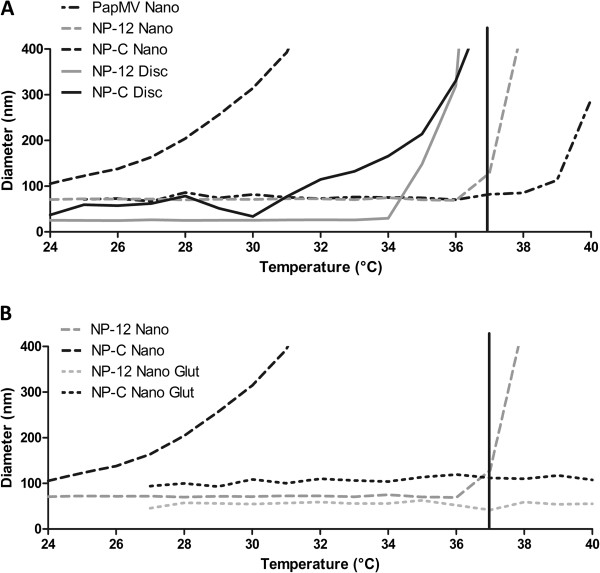
**Aggregation of PapMV nanoparticles and discs at different temperatures. A**) Aggregation state of recombinant nanoparticles or discs of PapMV NP-12, PapMV NP-C and PapMV (0.1 mg/ml) was measured by DLS at increasing temperature (speed of heating: 1°C/min.). The increase in diameter induced by heat is caused by the aggregation of nanoparticles. **B**) Using the same conditions as in (**A**), the aggregation of recombinant nanoparticles of PapMV NP-12, PapMV NP-C and PapMV either treated or not with glutaraldehyde was measured by DLS.

Fusion of a peptide to the PapMV vaccine platform could affect its stability, and potentially the ability to mount an immune response to the fused epitope [8]. As temperature can affect protein stability, we thus measured the influence of temperature on the aggregation of recombinant nanoparticles using DLS. Upon heating, proteins initiate partial denaturation through exposure of their hydrophobic residues to the solvent. This conformational change triggers formation of aggregates that can be measured easily by DLS. We found that PapMV NP-C nanoparticles initiated aggregation at 25°C while PapMV (without fusion) and NP-12 nanoparticles were more stable and initiated aggregation at 37°C or higher [Figure 3A]. Therefore, the higher stability of NP-12 nanoparticles at 37°C or higher appears to correlate with an optimal CTL response in mice.

**Figure 4:**
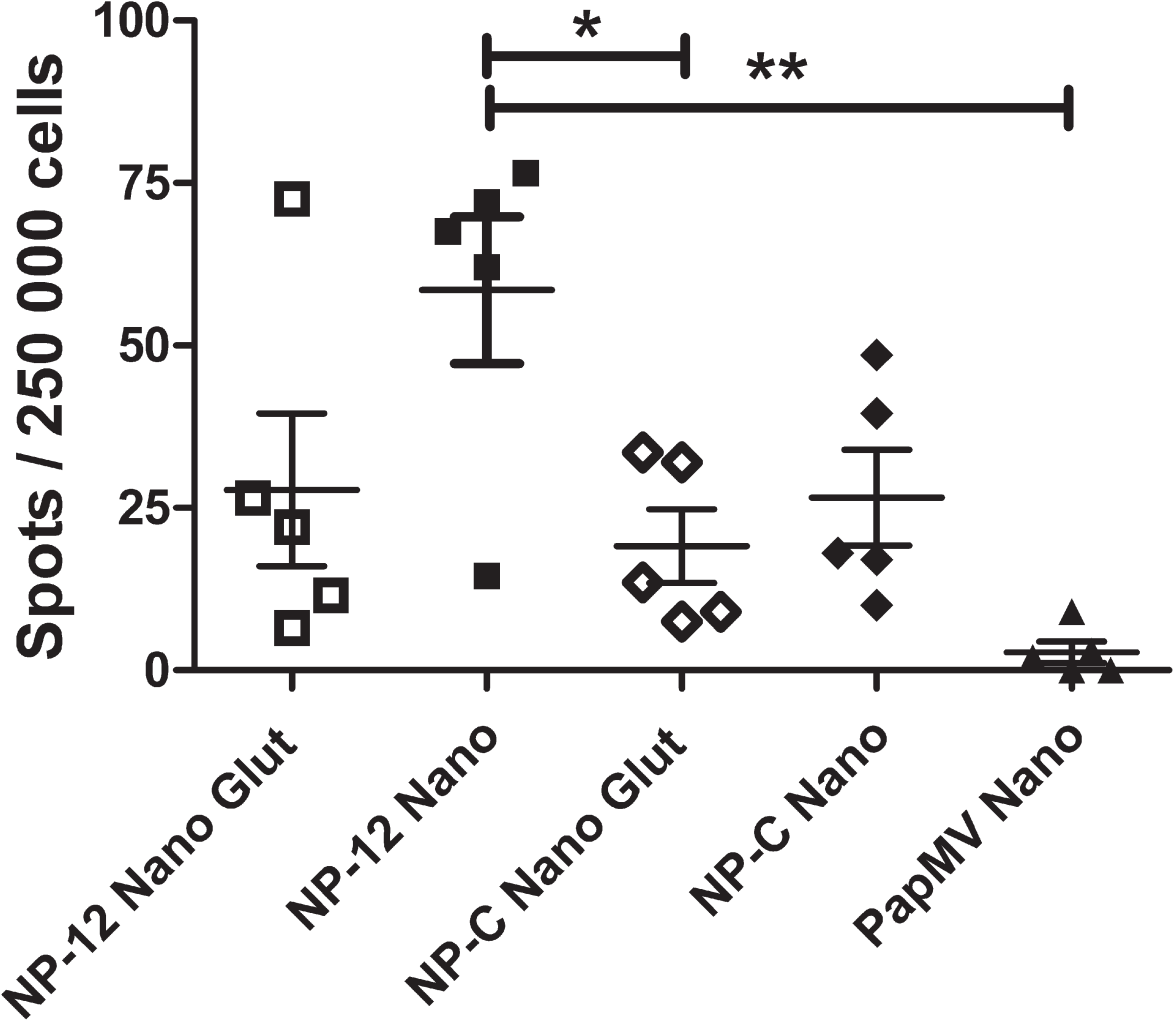
**ELISPOT assays of immunized mice. **Mice, 5 per group, were immunized three times by the intraperitoneal route with 100 μg of recombinant PapMV NP-12, PapMV NP-C and PapMV nanoparticles that were either untreated or cross-linked with glutaraldehyde. Two weeks after the second boost, spleens were extracted and ELISPOT assays performed. The precursor frequency of specific T cells was determined by subtracting the background spots in medium alone from the number of spots seen in wells reactivated with NP_147-155 _peptide. ** p ≤ 0.01 and * p ≤ 0.05.

**Figure 5:**
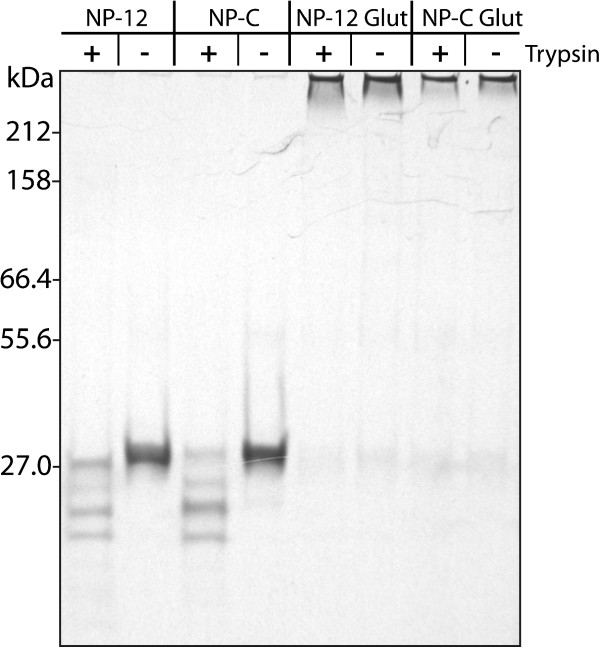
**Trypsin digests of recombinant PapMV NP nanoparticles cross-linked with glutaraldehyde. **PapMV NP-12 and PapMV NP-C either cross-linked with gluteraldehyde or not were submitted to a trypsin digest. The reaction was stopped by adding 10 μl of loading dye. Samples were heated for 10 minutes at 95°C prior to loading on an 8% SDS-PAGE.

Based on this observation, it was anticipated that stabilization of NP-C nanoparticles by chemical cross-linking should improve their immunogenicity. Therefore, we compared the capacity of NP-C and NP-12 nanoparticles either cross-linked with glutaraldehyde or not to induce a CTL response. The cross-linked nanoparticles were very stable even at temperatures exceeding 37°C [Figure 3B]. Mice (5 per group) were immunized three times at 2-week intervals with 100 μg of PapMV NP-C and NP-12 nanoparticles that were either cross-linked with glutaraldehyde or not. Surprisingly, cross-linking NP-C did not lead to an improved CTL response in mice [Figure 4]. The quantity of IFN-γ secreted by specific splenocytes remained similar to the response obtained with untreated nanoparticles. However, cross-linking of NP-12 nanoparticles did affect the efficacy CTL response induction as compared to native NP-12 [Figure 4]. We hypothesize that the cross-link made the nanoparticles too rigid, which consequently decreased their susceptibility to the proteases responsible for releasing the CTL epitope. To test this hypothesis, we resolved trypsin digests of cross-linked NP-12 and NP-C nanoparticles by SDS-PAGE (Figure 5). NP-12 and NP-C nanoparticles were only partially susceptible to the digest and lost 3 kDa on the gel as compared to non-digested nanoparticles. Cross-linked NP-C and NP-12 appeared resistant to digestion. The PapMV CP subunits of the cross-linked nanoparticles remained tightly attached to each other, leading to a very large molecular weight multimer that can barely enter the acrylamide gel. This experiment highlighted the likely difficulty of digesting cross-linked nanoparticles by host proteases involved in the excision of CTL epitopes for loading onto the MHC class 1 complex—an essential step in triggering a CTL response. In brief, nanoparticles must be sufficiently stable at animal body temperature (37°C) but flexible enough to allow their digestion by cellular proteases for loading onto MHC class 1 molecules. Therefore, an optimal balance between these two properties is crucial to triggering the CTL response efficiently.

### PapMV NP-12 nanoparticles but not discs are able to trigger a CTL response

In this experiment, our objective was to compare the efficacy of PapMV nanoparticles and discs in triggering a CTL response. PapMV NP-12 discs and nanoparticles were used to immunize (3 immunizations) mice (5 per group) with 100 μg of protein. We measured the production of IFN-μ after stimulation of splenocytes harvested from immunized mice 2 weeks after the second boost using the NP_147-155_ peptide. The level of IFN-γ secreted by splenocytes specific to PapMV NP-12 nanoparticles was significantly higher than that specific to PapMV NP-12 discs [Figure 2], suggesting that assembly into a highly ordered structure, i.e. nanoparticles, is critical to triggering the CTL response efficiently. We also noted that NP-12 discs appeared less stable than nanoparticles, and initiated aggregation at 34°C as compared to 37°C for nanoparticles [Figure 3]. Discs have the same diameter but are shorter than nanoparticles (30 nm vs 90 nm) and also less stable. These differences in size and stability could account for the observed differences in immunogenicity. Another difference between discs and nanoparticles is the RNA that they contain. Discs are associated with only very small amounts of RNA but nanoparticles contains ssRNA of bacterial origin [3]. It is possible that the ssRNA found in nanoparticles plays a role in the efficacy of the measured immune response. It is known that ssRNA of bacterial origin, as found in PapMV nanoparticles, can be recognized as pathogen associated molecular patterns (PAMPs) by several nucleic acid sensors like RIG-I, MDA-5, TLR7 or TLR8 that are at the interface between the innate and the adaptive immune response [15-17]. We are currently investigating if these sensors play a role in the CTL response.

Our results are consistent with findings obtained with peptide fusions made at the N-terminus of the CP of potato virus X (PVX)—another member of the potexvirus family. It was shown that production of recombinant PVX virus particles *in planta* can elicit either an humoral [18] or a CTL immune response [19]. The use of the N-terminus for fusion of peptides on this type of vaccine platform can, however, be problematic if the recombinant virus particles are produced in planta because the fusion may interfere with long-distance transport of the virus throughout the plants and thus affect yield [20]. This is one of the main reasons why we chose to produce our nanoparticles in a bacterial expression system that does not depend on the replication or cell-to-cell transport of the virus.

It is well accepted that the N-terminus of PapMV and potexvirus CP is exposed at the surface of the virus particle [2,8,21,22]. The recently published 3D structure of PapMV CP revealed that the N-terminus is involved in the interaction between two CP subunits in the virus particle [2], and that the 12 N-terminal residues upstream of F13 are directly exposed on the surface [14]. The availability of the CTL epitope located at this position, as compared to the C-terminus, probably facilitates its cleavage by host proteases and favours loading of the MHC class I pocket in the immune cells [6,9].

## Conclusions

The results of this study improve our understanding of the PapMV vaccine platform and highlight the importance of nanoparticle stability in triggering a CTL response. We can now beneficiate of two different points of fusion for a CTL epitope on the PapMV CP. The fusion at the N-terminus was clearly superior for the NP_147-155_ peptide but it does not mean that this will be the case for another CTL epitope. The amino acid sequence of the CTL epitope and its influence on the structure on the PapMV CP can have a major impact on its stability and their immunogenicity. Those results are increasing the versatility of the vaccine platform and provide more options for production of stable constructs. Because it is well established that the trigger of a CTL response to conserved epitopes is a valuable approach in the design of prophylactic or therapeutic vaccines to chronic diseases [23-27], we believe that the PapMV vaccine platform will be a very useful tool.

## Methods

### Ethics statement

All the work with animals adhered to the Institution-approved ethics protocol of the “Comité de Protection des Animaux” – CHUQ (CPA-CHUQ). The approval of this project is listed under the authorization number 2010148–1.

### Cloning and production of PapMV NP constructs

The PapMV CP construct (CPΔN5) used for this study has been described previously (Tremblay et al., 2006). To generate the PapMV NP constructs, PapMV CPΔN5 was used to introduce the following oligonucleotides by PCR at position 12 at the C-terminus of the coat protein [Table 1]. The linear vector harbouring the NP_147-155_ coding region fused to the PapMV was digested with BsiWI and ligated using T4 DNA ligase (New England BioLabs). The resulting PapMV NP clones harbour a fusion of the nucleoprotein epitope from Influenza virus at position 12 or at their C-terminus followed by a 6xHis tag for the purification process. We retained five amino acids on either side of the H-2Kd CTL epitope to ensure efficient processing. The integrity of the PapMV NP clones was confirmed by DNA sequencing. Expression and purification of PapMV particles fused to the NP_147-155_ peptide was performed as described previously [5]. Levels of expression for each recombinant nanoparticle were determined by SDS-PAGE. LPS contamination was always less than 50 endotoxin units (EU)/mg of protein. The size and structure of the nanoparticles were confirmed by observation on a TEM (JEOL-1010, Tokyo, Japan).

**Table 1:** Forward and reverse oligonucleotides used to produce PapMV CP recombinant proteins

***Name***	***Oligonucleotide sequence***
**NP-12**	
Forward	5^′^-AGCT***CGTACG***CGTGCGCTGGTTCGTACCGGTATGGACTTCCCCGCCATCACCCAGGAAC-3^′^
Reverse	5^′^-TCGA***CGTACG***CTGGTAGGTCGCGTCGTTCAGGTTGGCTATGTTGGGTGTGGATGCC-3^′^
**NP-C**	
Forward	5^′^-ACGT***CGTACG***CGTGCGCTGGTTCGTACCGGTATGGACACGCGTCACCATCACCATCAC-3^′^
Reverse	5^′^-TCGA***CGTACG***CTGGTAGGTCGCGTCGTTCAGGTTACTAGTTTCGGGGGG-3^′^

### Dynamic light scattering

The size of nanoparticles and discs was determined using a ZetaSizer Nano ZS (Malvern, Worcestershire, United Kingdom) at a temperature of 4°C and at a concentration of 0.1 mg/ml in PBS 1x for nanoparticles and at a concentration of 0.25 mg/ml in Tris–HCl 10 mM for discs. The thermal stability of PapMV nanoparticles was measured under the same experimental conditions at temperatures from 24°C to 40°C.

### Chemical cross-linking with glutaraldehyde

Cross-linking was performed using 0.1% glutaraldehyde in 10 mM Tris, 50 mM NaCl pH 7.5 in a final volume of 50 μl. The optimal concentration of protein for cross-linking was 150 ng/ml. After addition of glutaraldehyde, the mixture was incubated at room temperature for 30 minutes in the dark. The reaction was stopped with 15 μl of loading dye and heated for 10 minutes at 95°C before separating the proteins by 8% SDS-PAGE. The cross-linked proteins used to immunize mice were stored at 4°C until immunization without adding loading dye.

### SDS-PAGE and trypsin digest

Prior to SDS-PAGE, samples were mixed with one-third of the final volume of loading buffer containing 5% SDS, 30% glycerol and 0.01% bromophenol blue and heated for 10 minutes at 95°C. For the trypsin digest, we incubated 10 μg of proteins at 37°C in a volume of 50 μl for 120 minutes in 100 mM Tris–HCl pH 8.5 with 0.2 μg trypsin (Roche, 1418475). The reaction was stopped by adding 10 μl of loading dye. Samples were heated for 10 minutes at 95°C prior to loading on SDS-PAGE [3].

### Immunization

Five 6- to 8-week-old Balb/C mice (Charles River, Wilmington, MA) were immunized by the intraperitoneal route with: (i) 100 μg of PapMV NP-12 nanoparticles; (ii) 100 μg of PapMV NP-C nanoparticles; (iii) 100 μg of PapMV CP nanoparticles; (iv) 100 μg of PapMV NP-12 discs, and (v) 100 μg of PapMV NP-C discs. Primary immunization was followed by two booster doses given at 2-week intervals. Blood samples were obtained before each injection and 2 weeks after the last one and stored at -20°C until analysis.

### ELISPOT

Two weeks after the last boost, mice were sacrificed and spleens were recovered for ELISPOT assay performed as described previously [25]. The precursor frequency of specific T cells was determined by subtracting the background spots in media alone from the number of spots seen in wells reactivated with NP_147-155_ peptide. Data were analyzed with a parametric or a non-parametric ANOVA test when the variances differed significantly and with a Tukey or a Dunn post-test to compare difference among groups of mice. Values of *p < 0.05, **p < 0.01 and ***p < 0.001 were considered statistically significant. Statistical analyses were done with GraphPad PRISM 5.01.

## Competing interest

DL is founder and shareholder in the company FOLIA BIOTECH INC., a start-up company with the mandate to exploit commercially the PapMV nanoparticle technology. The patent is issued (U.S. Patent No. 7,641,896). This does not alter the authors’ adherence to all the BMC policies.

## Authors’ contributions

CB carried out all the experiments presented in this manuscript. NM and DL participated in the design of the study and supervision of CB. CB help to make the first draft of the manuscript. DL coordinated the study and completed the writing of the manuscript. All authors read and approved the final manuscript.

## References

[B1] Sit TL Abouhaidar MG Holy S Nucleotide sequence of papaya mosaic virus RNA J Gen Virol 1989 70 2325 2331 10.1099/0022-1317-70-9-2325 2778435

[B2] Yang S Wang T Bohon J Laliberté-Gagné ME Bolduc M Leclerc D Li H Crystal structure of the coat protein of the flexible filamentous papaya mosaic virus J Mol Biol 2012 422 263 273 10.1016/j.jmb.2012.05.032 22659319 PMC3418392

[B3] Tremblay MH Majeau N Laliberté-Gagné ME Lecours K Morin H Duvignaud J-B Bolduc M Chouinard N Paré C Gagné S Leclerc D Effect of mutations K97A and E128A on RNA binding and self assembly of papaya mosaic potexvirus coat protein FEBS J 2006 273 14 25 10.1111/j.1742-4658.2005.05033.x 16367744

[B4] Denis J Majeau N Acosta-Ramirez E Savard C Bedard M-C Simard S Lecours K Bolduc M Pare C Willems B Shoukry N Tessier P Lacasse P Lamarre A Lapointe R Lopez Macias C Leclerc D Immunogenicity of papaya mosaic virus-like particles fused to a hepatitis C virus epitope: Evidence for the critical function of multimerization Virology 2007 363 59 68 10.1016/j.virol.2007.01.011 17320136

[B5] Denis J Acosta-Ramirez E Zhao Y Hamelin M-E Koukavica I Baz M Abed Y Savard C Pare C Lopez Macias C Boivin G Leclerc D Development of a universal influenza A vaccine based on the M2e peptide fused to the papaya mosaic virus (PapMV) vaccine platform Vaccine 2008 26 3395 3403 10.1016/j.vaccine.2008.04.052 18511159

[B6] Leclerc D Beauseigle D Denis J Morin H Pare C Lamarre A Lapointe R Proteasome-independent major histocompatibility complex class I cross-presentation mediated by papaya mosaic virus-like particles leads to expansion of specific human T cells J Virol 2007 81 1319 1326 10.1128/JVI.01720-06 17121795 PMC1797532

[B7] Lacasse P Denis J Lapointe R Leclerc D Lamarre A Novel plant virus-based vaccine induces protective cytotoxic T-lymphocyte-mediated antiviral immunity through dendritic cell maturation J Virol 2008 82 785 794 10.1128/JVI.01811-07 17989184 PMC2224570

[B8] Rioux G Babin C Majeau N Leclerc D Engineering of papaya mosaic virus (PapMV) nanoparticles through fusion of the HA11 peptide to several putative surface-exposed sites PLoS One 2012 7 e31925 10.1371/journal.pone.0031925 22363771 PMC3283703

[B9] Hanafi L-A Bolduc M Gagné MEL Dufour F Langelier Y Boulassel M-R Routy J-P Leclerc D Lapointe R Two distinct chimeric potexviruses share antigenic cross-presentation properties of MHC class I epitopes Vaccine 2010 28 5617 5626 10.1016/j.vaccine.2010.06.024 20600515

[B10] Morin H Tremblay MH Plante E Paré C Majeau N Hogue R Leclerc D High avidity binding of engineered papaya mosaic virus virus-like particles to resting spores of Plasmodiophora Brassicae J Biotechnology 2007 128 423 434 10.1016/j.jbiotec.2006.10.013 17126944

[B11] Bachmann M Rohrer U Kundig T Burki K Hengartner H Zinkernagel R The influence of antigen organization on B cell responsiveness Science 1993 1993 262 1448 1451 8248784 10.1126/science.8248784

[B12] Jegerlehner A Storni T Lipowsky G Schmid M Pumpens P Bachmann MF Regulation of IgG antibody responses by epitope density and CD21-mediated costimulation Eur J Immunol 2002 32 3305 3314 10.1002/1521-4141(200211)32:11<3305::AID-IMMU3305>3.0.CO;2-J 12555676

[B13] Middelberg APJ Rivera-Hernandez T Wibowo N Lua LHL Fan Y Magor G Chang C Chuan YP Good MF Batzloff MR A microbial platform for rapid and low-cost virus-like particle and capsomere vaccines Vaccine 2011 29 7154 7162 10.1016/j.vaccine.2011.05.075 21651936

[B14] Laliberté Gagné ME Lecours K Gagné S Leclerc D The F13 residue is critical for interaction among the coat protein subunits of papaya mosaic virus FEBS J 2008 275 1474 1484 10.1111/j.1742-4658.2008.06306.x 18312419

[B15] Barbalat R Ewald SE Mouchess ML Barton GM Nucleic acid recognition by the innate immune system Annu Rev Immunol 2011 29 185 214 10.1146/annurev-immunol-031210-101340 21219183

[B16] Desmet CJ Ishii KJ Nucleic acid sensing at the interface between innate and adaptive immunity in vaccination Nat Rev Immunol 2012 12 479 491 10.1038/nri3247 22728526

[B17] Takeuchi O Akira S Pattern recognition receptors and inflammation Cell 2010 140 805 820 10.1016/j.cell.2010.01.022 20303872

[B18] Plchova H Moravec T Hoffmeisterova H Folwarczna J Cerovska N Expression of Human papillomavirus 16 E7ggg oncoprotein on N- and C-terminus of Potato virus X coat protein in bacterial and plant cells Protein Expr Purif 2011 77 146 152 10.1016/j.pep.2011.01.008 21266198

[B19] Lico C Mancini C Italiani P Betti C Boraschi D Benvenuto E Baschieri S Plant-produced potato virus X chimeric particles displaying an influenza virus-derived peptide activate specific CD8+ T cells in mice Vaccine 2009 27 5069 5076 10.1016/j.vaccine.2009.06.045 19563889

[B20] Lico C Capuano F Renzone G Donini M Marusic C Scaloni A Benvenuto E Baschieri S Peptide display on Potato virus X: molecular features of the coat protein-fused peptide affecting cell-to-cell and phloem movement of chimeric virus particles J Gen Virol 2006 87 3103 3112 10.1099/vir.0.82097-0 16963770

[B21] Chen TH Chen TH Hu CC Liao JT Lee CW Liao JW Lin MY Liu HJ Wang MY Lin NS Hsu YH Induction of protective immunity in chickens immunized with plant-made chimeric bamboo mosaic virus particles expressing very virulent infectious bursal disease virus antigen Virus Res 2012 166 109 115 10.1016/j.virusres.2012.02.021 22406128

[B22] Rioux G Majeau N Leclerc D Mapping the surface-exposed regions of papaya mosaic virus nanoparticles FEBS J 2012 279 2004 2011 10.1111/j.1742-4658.2012.08583.x 22524169

[B23] Leclerc D Plant viral epitope display systems for vaccine development Curr Top Microbiol Immunolin press 10.1007/82_2011_183 22021043

[B24] Gandhi RT Walker BD Immunologic control of HIV-1 Annu Rev Med 2002 53 149 172 10.1146/annurev.med.53.082901.104011 11818468

[B25] Yerly D Heckerman D Allen TM Chisholm JV Faircloth K Linde CH Frahm N Timm J Pichler WJ Cerny A Brander C Increased cytotoxic T-lymphocyte epitope variant cross-recognition and functional avidity are associated with hepatitis C virus clearance J Virol 2008 82 3147 3153 10.1128/JVI.02252-07 18184704 PMC2258967

[B26] Carragher DM Kaminski DA Moquin A Hartson L Troy DR A novel role for Non-neutralizing antibodies against nucleoprotein in facilitating resistance to influenza virus J Immunol 2008 181 4168 4176 18768874 10.4049/jimmunol.181.6.4168 PMC2590646

[B27] Savard C Laliberté-Gagné ME Bolduc M Guérin A Drouin K Forget MA Majeau N Lapointe R Leclerc D Improvement of the PapMV nanoparticle adjuvant property through an increased of its avidity for the antigen (Influenza NP) Vaccine 2012 15 2535 2542 22326774 10.1016/j.vaccine.2012.01.085

